# Diagnosis and Treatment of Spinal Syphilitic Gumma: A Case Report

**DOI:** 10.3389/fneur.2019.01352

**Published:** 2020-01-15

**Authors:** Linyang Cui, Zushan Xu, Hongjun Hou

**Affiliations:** Department of Radiology, Weihai Central Hospital, Weihai, China

**Keywords:** neurosyphilis, spinal syphilitic gumma, magnetic resonance imaging, diagnosis, anti-syphilitic treatment

## Abstract

Neurosyphilis, which is caused by *Treponema pallidum*, is a rare sexually transmitted disease involving the central nervous system (CNS). Among all the sub-categories, spinal syphilitic gumma is extremely rare. In previous literature, limited cases of spinal syphilitic gumma have been reported, most of which underwent surgery treatment. In this study, we reported a 46-year-old man, who presented with 5-day numbness of bilateral lower limbs, lower back pain, and irregular defecation. Magnetic resonance imaging (MRI) revealed a homogeneous peripheral enhancement and intramedullary nodule at the T7 level with extensive thoracic cord edema. Combining with laboratory examination results, the syphilitic gumma was considered. One month after the administration of penicillin G, the symptoms vanished. Six months later, MRI indicated no intramedullary nodule.

## Introduction

Syphilis is a chronic infectious disease caused by *Treponema pallidum*. The incidence of syphilis has dramatically declined since the availability of penicillin treatment. However, a remarkable increasing incidence has been suggested in recent reports, especially among human immunodeficiency virus (HIV)-positive patients and homosexual men (1, 2). Syphilis can affect various organ systems, including the spinal cord. It is noteworthy that spinal syphilitic gumma is exceedingly rare in clinical practice, which could be misdiagnosed as tumors.

## Case Report

**Figure 1:**
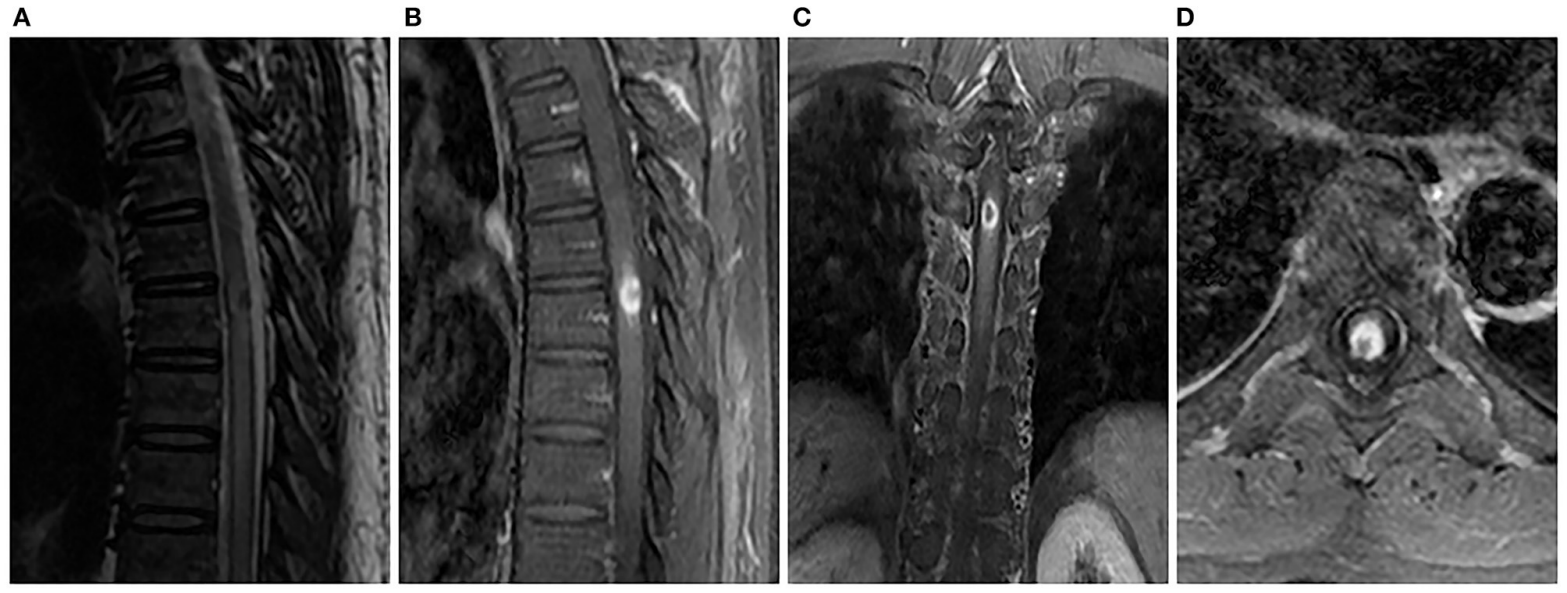
Spinal MRI showed an intramedullary heterogeneous nodule at the T5 level that was associated with extensive thoracic cord edema, while most hyperintense was associated with a marked hypointense core on sagittal T2-weighted image **(A)**. Sagittal **(B)**, coronal **(C)**, and axial **(D)** T1-weighted images with contrast revealed obvious peripheral enhancement and no central enhancement.

**Figure 2:**
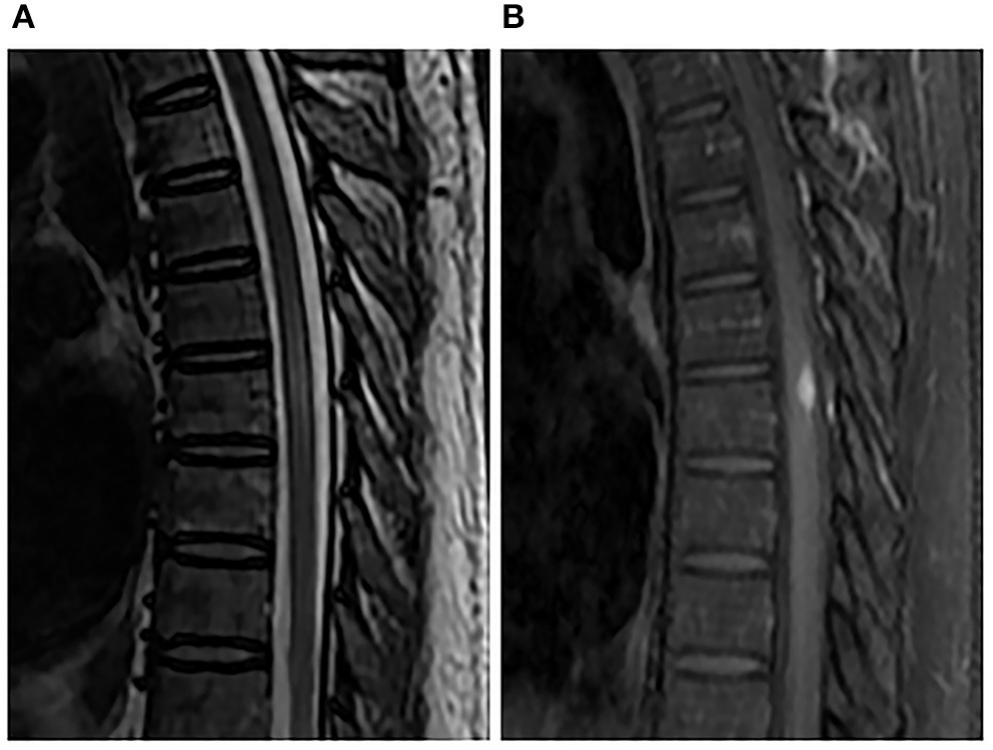
Spinal MRI performed 1 month after penicillin therapy. Sagittal T2-weighted image **(A)** and enhanced T1-weighted image **(B)** showed that the nodule and perilesional edema were reduced.

**Figure 3:**
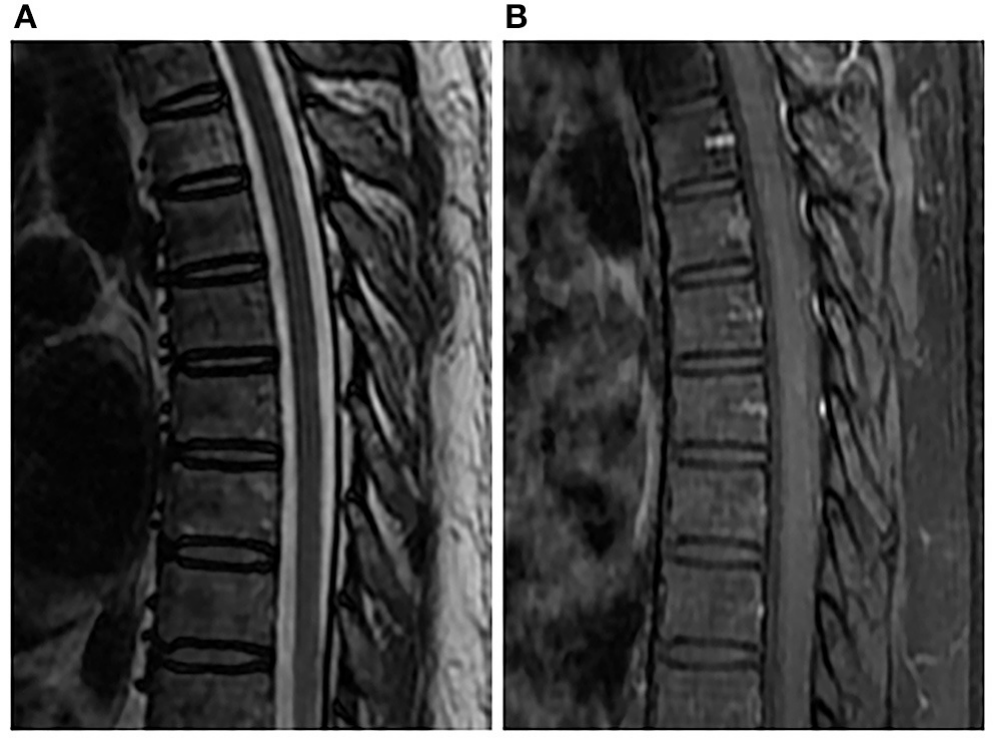
Spinal MRI performed 6 months after penicillin therapy. Sagittal T2-weighted image **(A)** and enhanced T1-weighted image **(B)** showed normal spinal cord.

A 46-year-old male worker was admitted to the hospital with the symptoms of bilateral lower-limb numbness, lower back pain, and irregular defecation for 5 days. The pain gradually spread to the upper abdomen. Physical examination demonstrated muscle strength of grade 4/5 in lower extremities and grade 5 in upper extremities. There was a presence of hyperalgesia below the T7 level. Muscle tension was normal. Further neurologic examinations revealed normal deep tendon reflexes in arms and legs, as well as normal abdominal reflexes. Pathologic reflexes were also negative on both sides. In addition, the patient had a history of hypertension medication and kidney failure in compensated period. The patient had no history of trauma, cancer, diabetes, or allergic diseases. MRI of the spinal cord demonstrated swelling of the thoracic cord with long-segment diffuse high signal intensity and a heterogeneous nodule with hypointense center at the T7 level on T2-weighted imaging. The post-gadolinium imaging indicated peripheral enhancement of the nodule in the dorsal aspect of spinal cord, while no enhancement of the diffuse high-signal lesion was observed on the T2-weighted images (Figures 1A–D). Brain MRI was normal. Performance of MRI may indicate spinal tumors or inflammatory granuloma such as syphilis, tuberculosis, and neurocysticercosis. Cerebrospinal fluid (CSF) examination showed elevated cell (120/μl, 76% lymphocytes) and protein levels (84 mg/dl), while the levels of glucose and chloride were normal. Syphilis serology including the *Treponema pallidum* particle agglutination (TPPA) and toluidine red unheated serum test (TRUST) demonstrated positive results. The TRUST titer of serum and CSF were 1/128 and 1/32, respectively. Serological test for HIV was negative. Normal results were observed in the tests for AQP4-IgG, antinuclear antibody, rheumatoid factor, tuberculosis antibody, neurocysticercosis antibody, and tumor marker. The patient denied having a history of venereal diseases and exposure to unprotected intercourse with commercial sexual workers or homosexuality, and also denied any previous symptoms relevant to syphilis infection. There were no skin or mucous lesions or chancre at present. However, given the results of syphilis serologic test, CSF examination, and MRI scan, spinal syphilitic gumma was strongly suspected. The patient was treated with penicillin G (24 million U/day intravenously every 6 h for 14 days) and prednisolone (20 mg/day for 3 days). Three days after the treatment, back pain and bilateral lower-limb numbness were obviously lessened, and irregular defecation was changed correctly. One month after the onset, spinal MRI showed that the lesion was reduced compared with that before the treatment (Figures 2A,B), and the result of the CSF routine test was approaching normal. Serum and CSF TPPA were positive, and TRUST titer of serum and CSF were 1/4 and 1/1, respectively. After 6-month follow-up, the symptoms of pain and numbness disappeared, and CSF studies and spinal MRI demonstrated normal results (Figures 3A,B). A definitive diagnosis of spinal syphilitic gumma was made based on the clinical symptoms, MRI findings, and laboratory tests, as well as with the favorable prognosis after the penicillin therapy.

## Discussion

Neurosyphilis (NS) is observed in 4–10% of patients with untreated or insufficiently treated syphilis (3), which could develop at any stage of the disease. Compared with the intracerebral syphilis, spinal syphilis is relatively rare, mainly including myelitis, myelophthisis, and gumma. Although extremely low in prevalence, spinal syphilitic gumma is a strong inflammatory response in which *T. pallidum* invades the spinal cord from the meninges and vessels, which may cause severe outcomes.

Spinal syphilitic gumma can occur in intramedullary, intradural-extramedullary, or extradural space, which have different appearance in the results of imaging (4). However, previous spinal and cerebral gumma cases also reported a few common imaging characteristics (4–6), including rounded lesion, which is surrounded by extensive edema, and caseous necrosis center with low signal or mixed normal and low signal on T2-weighted imaging. Besides, distinct enhancement in the periphery of the nodule could be observed in the gadolinium-enhanced MRI. The signal characteristics in MRI are related to the pathologic tissue structure of gumma, which is a granulomatous inflammation with a cheese-like necrotic core and surrounded by lymphocytes epitheloid cells and Langhans giant cells. The low signal foci of caseous necrosis on T2-weighted imaging are due to the paramagnetic free radical produced by the macrophages. Although meningeal involvement was believed to be a characteristic sign of cerebral parenchyma gumma, it was not observed in the three cases of entirely intramedullary gumma reported so far (including this case) (4, 7). Thus, the role of meningeal involvement in syphilitic gumma should be verified by further research. Our spinal MRI showed an intramedullary nodule at the T7 level that was associated with extensive thoracic cord edema. The heterogeneous nodule was presented as slightly hypointense to isointense on T1-weighted imaging, while most hyperintense were associated with a marked hypointense core on T2-weighted imaging. After contrast administration, the peripheral portion of lesion was apparently enhanced, and the low signal core was not enhanced. Eventually, a clear diagnosis of spinal syphilitic gumma was made after combining with the laboratory test results. The differential diagnosis mainly includes tuberculosis, sarcoidos, neurocysticercosis, and spinal tumors, for which similar imaging manifestations could also be observed, such as irregular annular enhancement nodular lesion and low signal on T2-weighted imaging at the center portion and surrounding edema to different extent.

A series of comprehensive information are needed to be integrated before the diagnosis of NS, such as patient history, clinical manifestations, imaging, and serum and CSF tests such as TPPA, TRUST, or Venereal Disease Research Laboratory (VDRL). Among laboratory tests, a positive CSF VDRL test has proven to be the most highly specific diagnostic criterion, but with low sensitivity. Though the spinal syphilitic gumma in our case was not confirmed by operation pathology, the supportive laboratory tests and MRI findings in the patient could prompt diagnosis.

The recommended treatment for patients with NS is intravenous injection of aqueous penicillin G at 18–24 million U/day for 10–14 days. For patients allergic to penicillin, intravenous injection of Ceftriaxone (2 g/day) could be considered as an acceptable alternative. Prednisolone is routinely added to prevent cord edema or Jarisch-Herxheimer reactions before the start of penicillin. After the systemic anti-syphilis treatment, the symptoms of syphilitic gumma disappeared in our case. As it is extremely rare in clinical practice, almost all of the previously reported spinal syphilitic gumma cases were suspected as spinal tumors and were subjected to the surgery (4, 7–14). Instead, most of the cerebral syphilitic gumma in the literature can be significantly reduced or completely absorbed after the anti-syphilis treatment, and surgery would be unnecessary (15). To our knowledge, this is the second case report of spinal syphilitic gumma that was treated with a non-surgical treatment (16). Surgery is surely an optimal treatment for the patients with acute spinal cord compression or whose gumma cannot be completely cured by anti-syphilis therapy. However, high-dose penicillin therapy should be recommended first, rather than surgical treatment, for patients without neurologic deterioration who are diagnosed with NS (including the spinal syphilitic gumma) through analysis of imaging findings and laboratory examinations.

Spinal syphilitic gumma is an extremely rare manifestation of NS. Certain common MRI characteristics may contribute to the diagnosis of spinal syphilitic gumma in a syphilis-infected patient. After the diagnosis was made, instead of surgical treatment, anti-syphilitic treatment could be used to reverse the disease.

## Ethics Statement

This study was approved by the Ethics Committee of Weihai central hospital. Written informed consent was obtained from the patient for the publication of this case report.

## Author Contributions

LC contributed to the data collection and writing. HH and ZX guided the completion of this article. All authors contributed to manuscript revision, read, and approved the submitted version.

### Conflict of Interest

The authors declare that the research was conducted in the absence of any commercial or financial relationships that could be construed as a potential conflict of interest.
